# Novel CD19 chimeric antigen receptor T cells manufactured next-day for acute lymphoblastic leukemia

**DOI:** 10.1038/s41408-022-00688-4

**Published:** 2022-06-24

**Authors:** Cheng Zhang, Jiaping He, Li Liu, Jishi Wang, Sanbin Wang, Ligen Liu, Jian Ge, Lei Gao, Li Gao, Peiyan Kong, Yao Liu, Jia Liu, Yu Han, Yongliang Zhang, Zhe Sun, Xun Ye, Wenjie Yin, Martina Sersch, Lianjun Shen, Wei William Cao, Xi Zhang

**Affiliations:** 1 Medical Center of Hematology, Xinqiao Hospital, State Key Laboratory of Trauma, Burn and Combined Injury, Army Medical University, Chongqing, P. R. China; 2 Gracell Biotechnologies Ltd, Shanghai, P. R. China; 3 Department of Hematology, Tangdu Hospital, Air Force Medical University, Xi’an, Shaanxi P. R. China; 4 Department of Hematology, The Affiliated Hospital of Guizhou Medical University, Guiyang, Guizhou P. R. China; 5 Department of Hematology, 920th Hospital of Joint Logistics Support Force, Kunming, Yunnan P. R. China; 6 Department of Hematology, Tongren Hospital, Shanghai Jiao Tong University School of Medicine, Shanghai, P. R. China; 7 Department of Hematology, The First Affiliated Hospital of Anhui Medical University, Hefei, Anhui P. R. China

**Keywords:** Phase I trials, Cancer immunotherapy

## Abstract

Chimeric antigen receptor-engineered T (CAR-T) cells have shown promising efficacy in patients with relapsed/refractory B cell acute lymphoblastic leukemia (R/R B-ALL). However, challenges remain including long manufacturing processes that need to be overcome. We presented the CD19-targeting CAR-T cell product GC007F manufactured next-day (FasTCAR-T cells) and administered to patients with R/R B-ALL. A total of 21 patients over 14 years of age with CD19^+^  R/R B-ALL were screened, enrolled and infused with a single infusion of GC007F CAR-T at three different dose levels. The primary objective of the study was to assess safety, secondary objectives included pharmacokinetics of GC007F cells in patients with R/R B-ALL and preliminary efficacy. We were able to demonstrate in preclinical studies that GC007F cells exhibited better proliferation and tumor killing than conventional CAR-T (C-CAR-T) cells. In this investigator-initiated study all 18 efficacy-evaluable patients achieved a complete remission (CR) (18/18, 100.00%) by day 28, with 17 of the patients (94.4%) achieving CR with minimal residual disease (MRD) negative. Fifteen (83.3%) remained disease free at the 3-month assessment, 14 patients (77.8%) maintaining MRD negative at month 3. Among all 21 enrolled patients, the median peak of CAR-T cell was on day 10, with a median peak copy number of 104899.5/µg DNA and a median persistence period of 56 days (range: 7–327 days). The incidence of cytokine release syndrome (CRS) was 95.2% (*n* = 20), with severe CRS occurring in 52.4% (*n* = 11) of the patients. Six patients (28.6%) developed neurotoxicity of any grade. GC007F demonstrated superior expansion capacity and a less exhausted phenotype as compared to (C-CAR-T) cells. Moreover, this first-in-human clinical study showed that the novel, next-day manufacturing FasTCAR-T cells was feasible with a manageable toxicity profile in patients with R/R B-ALL.

## Introduction

Chimeric antigen receptor-engineered T cells (CAR-T cells) represent a major advance in cancer treatment [1–6]. To date, three CAR-T cell products directed against the antigen CD19 have been approved for the treatment of hematological malignancies in the United States [7, 8]. However, challenges remain. In two large-scale CD19-directed CAR-T cell clinical trials, 20–30% of candidates were ultimately not infused with CAR-T cells due to manufacturing failures and/or disease progression during manufacturing [9, 10]. The manufacturing time for conventional CAR-T (C-CAR-T) cells is currently 9-14 days, and the vein-to-vein time ranges from 3-4 weeks [9, 10]. The longer manufacturing period and therefore wait time may allow existing malignancies to progress, which can result in worse outcomes and more adverse events [11, 12]. The extensive ex vivo culture needed to produce CD19-directed CAR-T cells may result in a significant loss of T cell stemness, which limits engraftment and persistence following adoptive transfer and decreases therapeutic effectiveness [13, 14].

In this study, patients were treated with a novel type of CAR-T cells manufactured on a novel manufacturing platform (FasTCAR) with next-day manufacturing - GC007F cells. Through a series of in vitro and in vivo preclinical experiments, we compared the novel GC007F cells to C-CAR-T cells in regard to expansion capability, phenotype, and potency in eliminating B-ALL. Based on the results of these preclinical studies, a multicenter clinical study was initiated to determine the safety and efficacy of CD19-directed GC007F cells in treating patients with CD19^+^ relapsed/refractory B cell acute lymphoblastic leukemia (R/R B-ALL).

## Methods

### Genetic constructs

The mouse FMC63 anti-CD19 single-chain variable fragment (scFv) was inserted into a second-generation CAR cassette containing a CD8α signal peptide, a CD8α hinge region, a CD8α transmembrane domain, a CD28 costimulatory domain, and a CD3ζ activation domain. This CAR construct is herein called CD19-CAR. A FLAG tag (DYKDDDDK) was inserted into CD19-CAR between the scFv and the hinge region for detection by flow cytometry.

### GC007F cell production with the FasTCAR platform

T cells were isolated, and GC007F cells and C-CAR-T cells were generated as previously reported [15]. Peripheral blood mononuclear cells (PBMCs) were obtained from healthy donors (preclinical studies) and enrolled patients (clinical study) by leukapheresis using COM. TEC (Fresenius Kabi) or COBE Spectra (Terumo BCT). T cells were isolated using Dynabeads CD3/CD28 CTS (Thermo Fisher Scientific) according to the manufacturer’s instructions and transduced with lentiviral vectors carrying the CD19-CAR construct in X-VIVO 15 medium (Lonza). Lentiviral vectors were added at a multiplicity of infection (MOI) of 1–5, and cell-vector mixtures were incubated overnight in an incubator at 37 °C with 5% CO_2_.

### CAR-T cell characterization

To evaluate the proliferation of GC007F cells, frozen GC007F cells and C-CAR-T cells were thawed, cultured for 2 days in porous (Corning) plates or flasks and then stimulated with irradiated K562-CD19 cells every 3 days. During expansion, cultured CAR-T cells were split, and the cell viability and number were examined using the NC-200^™^ Automated Cell Counter according to the manufacturer’s instructions. The absolute number of CAR-T cells during expansion was calculated based on the percentage of CAR-positive cells determined using flow cytometry three times. To compare the stemness and memory features of GC007F cells and C-CAR-T cells, flow cytometry was used to detect the expression of CD45RO and CD62L. Central memory T cells (T_CM_ cells) were defined as CD62L^+^ CD45RO^+^  cells, and effector memory T cells (T_EM_ cells) were defined as CD62L^-^CD45RO^+^  cells. To detect the exhaustion of GC007F cells and C-CAR-T cells, flow cytometry was used to detect the expression of PD-1, Tim3 and LAG3.

### In vitro cytotoxicity assay

A cytotoxicity assay was performed on an xCELLigence RTCA instrument (ACEA Biosciences) according to the manufacturer’s instructions. Briefly, HeLa-CD19 cells were cocultured with GC007F cells and C-CAR-T cells at various effector-to-target (E/T) ratios in triplicate, and growth inhibition was monitored; the experiment was repeated three times. In a second cytotoxicity assay, luciferase-expressing Nalm6 cells were used as target cells. CAR-T cells and target cells were cocultured in 96-well plates at various effector-to-target ratios for 6 h, and the coculture supernatant was harvested and frozen for further cytokine assays. Surviving target cells were quantified using the Promega One-Glo Luciferase Assay System according to the manufacturer’s instructions. The percent killing was calculated as [(Target cell only wells-CAR-T cell treatment wells)/Target cell only wells] × 100%.

### Cytokine assay

The production of the cytokines interferon-γ (IFN-γ) and interleukin (IL)-2 by cultured cells was quantified. The levels of serum cytokines, chemokines and growth factors in clinical samples were measured according to the manufacturer’s instructions. Protocol details are provided in the supplementary material.

### Animal model

Animal studies were performed under protocols approved by the Institutional Animal Care and Use Committees (IACUCs) of the service providers. NOG mice were injected intravenously (i.v.) with Nalm6-Luciferase cells (1 × 10^6^/mouse), followed by intravenous infusion of various doses of CD19-targeted CAR-T cells 7 days later. Tumor growth and mouse survival were monitored.

### Study design and participants

The clinical study was designed to evaluate the safety and efficacy of three different doses of CAR-T cells (dose level (DL) 1: 0.5 × 10^5^ CAR^+^ T cells/kg, DL2: 1.0 × 10^5^ CAR^+^ T cells/kg, and DL3: 1.5 × 10^5^ CAR^+^ T cells/kg) in patients over 14 years with CD19^+^ R/R B-ALL. Major inclusion criteria included (1)Male and female patients aged 14 to 70 years were eligible; (2) Patients were diagnosed with R/R B-ALL with any one of the following conditions: (a) relapse, including two or more relapses or relapse after allogeneic hematopoietic stem cell transplantation (allo-HSCT) with 5% blast cells; (b) refractory disease, including no complete remission (CR) or continuous minimal residual disease (MRD) positivity after two cycles of chemotherapy with gene mutation associated with unfavorable prognosis or with complex karyotype; (c) Philadelphia chromosome (Ph)-positive B-ALL with any one of the following conditions: failure to tolerate tyrosine kinase inhibitors (TKIs), progression after the first and second TKI treatments or unfit for allo-HSCT; (3) Participants with an ECOG performance status ≤1 at study entry were eligible. (4) Patients with an estimated survival time of over three months and (5) adequate main organ function were eligible. The study was approved by the Ethics Committee of the Xinqiao Hospital, and it was conducted according to Good Clinical Practice China and the principles of the Declaration of Helsinki (ChiCTR1900023212). All patients provided written informed consent prior to participation in any study procedures.

### GC007F cell therapy and pharmacokinetics

After peripheral blood mononuclear cell (PBMC) (3–5 × 10^9^) collected by leukapheresis, the eligible enrolled patients would receive GC007F cell infusion following the FC conditioning regimen (30 mg/m^2^/day fludarabine and 300 mg/m^2^/day cyclophosphamide over 2 through 5 days), and then pharmacokinetics were examined. The conditioning regimens were used depending on the blasts percentage, with two days for <5%, three days for 5%−20%, four days for 20%−50%, and five days for over 50%. Bone marrow was collected to evaluate blasts by flow cytometry. Patient response after treatment was assessed according to the National Comprehensive Cancer Network (NCCN) guidelines “Acute Lymphocytic Leukemia Remission Criteria” 2016 V2 edition for treatment response in B-ALL.

**Fig. 1:**
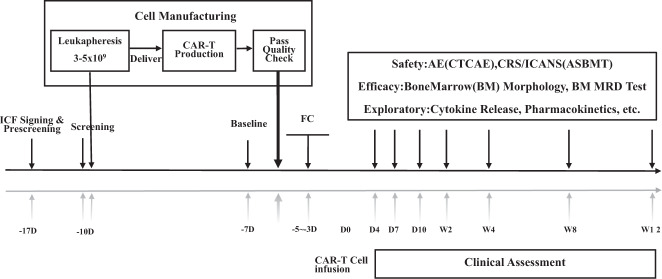
Clinical study schema. After screening the patients and signing informed consent form, the PBMCs were collected by leukapheresis. The GC007F cells were constructed and infused into patients after conditioning regimens. The pharmacokinetics, response assessment and adverse events were observed. ICF Informed consent form, PBMCs peripheral blood mononuclear cells, MRD minimal residual disease, AE advert effect, CRS cytokine release syndrome, ICANS immune effector cell-associated neurotoxicity syndrome, CTCAE common terminology criteria for adverse events, ASBMT American society for blood and marrow transplantation.

After GC007F cell infusion, PBMCs were collected to evaluate cell pharmacokinetics on days 4, 7, and 10 and weeks 2, 4, 8 and 12; assessment were performed using qPCR and flow cytometry. Bone marrow samples were collected for evaluation of blasts by flow cytometry. The clinical study schema was showed in Fig. 1.

### Response assessment

A blast load less than 5% in the bone marrow was defined as complete remission (CR). A bone marrow blast level below 0.01% assessed using multiparameter flow cytometry or 0% BCR/ABL detected by qPCR for Ph-positive B-ALL was defined as minimal residual disease (MRD) negative. The reappearance of MRD positivity or blasts in the blood, bone marrow or an extramedullary site after CR was defined as relapsed disease. Bone marrow was evaluated every month after infusion of GC007F cells.

### Adverse events

The severity of cytokine release syndrome (CRS) in patients presenting with fever, myalgia, hypotension, hypoxia or organ toxicity was analyzed using the American Society for Blood and Marrow Transplantation (ASBMT) consensus grading system [16]. CRS was considered severe if it was of grade 3 or higher. Neurotoxic effects, including aphasia, altered level of consciousness, impairment in cognitive skills, motor weakness, seizures, and cerebral edema, were based on the Common Terminology Criteria for Adverse Events (CTCAE) 4.0.3. A seizure of any grade or a neurotoxic effect of grade 3 or higher was defined as a severe neurotoxic effect. CRS and neurotoxic effects were managed according to standard management guidelines.

### Statistics

Data are presented as mean ± standard deviation or median (minimum-maximum) for continuous variables and number (%) of patients for categorical variables as appropriate. Analysis of variance or Wilcoxon rank sum test was used to compare the characteristics between GC007F cells and C-CAR-T cells. The Kaplan-Meier survival curves were drawn for progression free survival (PFS) and overall survival (OS), respectively. PFS is defined as the time interval from the beginning of GC007F infusion to disease recurrence or death from any cause. OS is defined as the time interval from the beginning of GC007F infusion to death of any cause. The primary objective of the study was to assess the safety and pharmacokinetics of GC007F cells in patients with R/R B-ALL. The secondary objective was to assess efficacy, including CR and duration of response. The median follow-up time was 8 months (range, 2–29 months).

A swimmers plot was provided to display effects of the study treatment on tumor response for individual patients. For serum cytokine data, univariate linear regression model was used to explore their relationship with tumor load or CAR-T cell count or Wilcoxon rank sum test with CRS or neurotoxic effects of grade 3 or above. Additional bar plots or line graphs over nominal time were provided as appropriate. The demographic and baseline characteristics, efficacy data was analyzed based on full analysis set and safety data using safety set. *P* values less than 0.05 at two-sided were considered statistically significant. All the statistical analyses were performed using R 4.0.3 (R Development Core Team), Stata 16.0 (Stata Corp), or GraphPad Prism 9.0 (GraphPad Software).

## Results

### Characteristics of GC007F cells

**Fig. 2:**
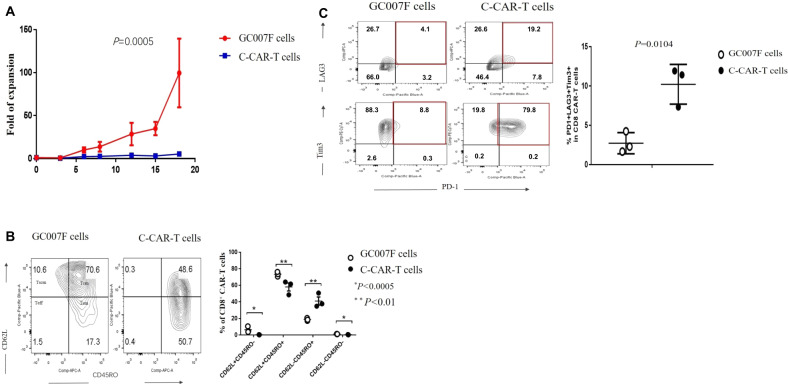
Characteristics of GC007F cells. Proliferation-, phenotype-, and expression-related cell-surface markers were examined by flow cytometry. **A** Proliferation of GC007F cells. **B** T cell memory and stemness features were characterized by surface staining of GC007F cells and C-CAR-T cells for CD45RO and CD62L and evaluation using flow cytometry (left) and statistical analysis (right). CD62L^+^CD45RO^+^ central memory T cells (T_CM_ cells) and CD62L^-^CD45RO^+^ effector memory T cells (T_EM_ cells). **C** T cell exhaustion was characterized by surface staining for PD-1, LAG3 and Tim3, which was analyzed using flow cytometry (left) with statistical analysis of the % of PD1^+^LAG3^+^Tim3^+^ cells (right).

GC007F cells showed significantly greater expansion than C-CAR-T cells (*P* = 0.0005) (Fig. 2A). We further examined differences in T cell stemness and memory phenotypes, as previously reported [17, 18]. As shown in Fig. 2B, C-CAR-T cells consisted of relatively comparable frequencies of central memory T cells (T_CM_ cells; CD62L^+^CD45RO^+^, 58.03% ± 8.34%) and effector memory T cells (T_EM_ cells; CD62L^-^CD45RO^+^, 41.06% ± 8.47%). In contrast, T_CM_ cells (73.47% ± 2.85%) predominated among GC007F cells, followed by T_EM_ cells (18.8% ± 1.77%). GC007F cells had a higher T_CM_ cell frequency (*P* < 0.05) and a lower T_EM_ cell frequency (*P* < 0.01) than C-CAR-T cells, which indicated that GC007F cells were generally in an earlier stage of T cell differentiation and had a younger phenotype.

The proportion of cells expressing the immune checkpoint molecules PD-1, LAG3 and Tim3 was upregulated in exhausted C-CAR-T cells. In marked contrast, GC007F cells exhibited a significantly lower proportion of positive cells for these exhaustion markers (*P* = 0.0104) (Fig. 2C).

### GC007F cells have a potent in vitro killing capacity

**Fig. 3:**
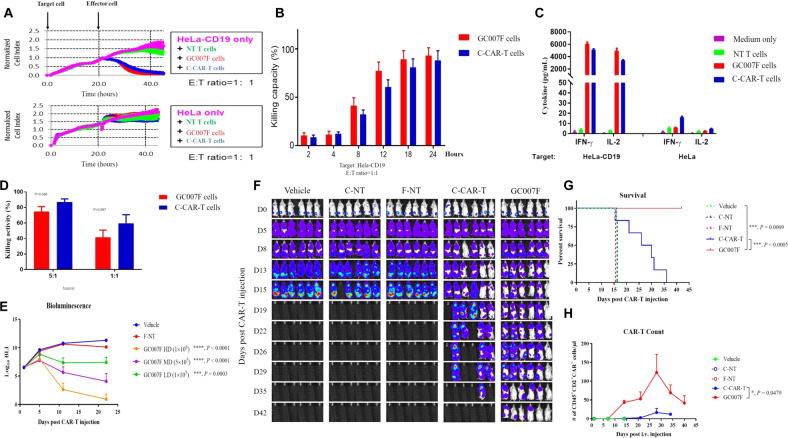
In vivo and in vitro tumoricidal effects of GC007F cells. **A** The specific killing of HeLa-CD19 cells was detected by RTCA assays. **B** Growth curves for HeLa-CD19 cells in the GC007F cell- and C-CAR-T cell-treated groups. **C** The concentrations of IFN-γ and IL-2 in the culture supernatant were quantified using ELISA. **D** CD19^+^  Nalm6-luciferase cells were cocultured with CAR-T cells at different ratios, and the cell-killing efficacy (%) was determined by measuring luciferase activity. **E** The tumor burdens in mice treated with different doses of GC007F cells were measured using IVIS. HD: high dose, MD: medium dose, LD: low dose. **F** Bioluminescence imaging of the tumor burden at the indicated time points after CAR-T cell infusion (5 × 10^5^ cells). **G** Survival of tumor-bearing mice treated with GC007F cells, C-CAR-T cells, or the corresponding nontransduced (NT) controls (C-NT or F-NT cells). **H** The expansion of infused CD45^+^CD2^+^CAR^+^ T cells in the peripheral blood was quantitated using flow cytometry. Data are representative of two or more independent experiments. All data represent the mean ± SEM (*n* ≥ 6 per group) and were analyzed using two-way ANOVA (**E**, versus vehicle), the Mantel-Cox test (**G**), or a paired *t* test (**H**).

GC007F cells and C-CAR-T cells selectively killed HeLa-CD19 cells but not HeLa cells, exhibiting comparable cytotoxicity (Fig. 3A). This result indicated that the CD19-CAR construct specifically targeted CD19. Similar growth curves for HeLa-CD19 cells were observed in the GC007F cell- and C-CAR-T cell-treated groups (Fig. 3B). Consistently, similar production of IFN-γ and IL-2 was detected for GC007F cells and C-CAR-T cells (Fig. 3C).

GC007F cells showed a lower in vitro killing capacity than C-CAR-T cells at a ratio of 5:1 (Fig. 3D) and a similar killing capacity at a ratio of 1:1 in a luciferase-based assay. The lower killing capacity of GC007F cells might be related to the low percentage of TEM cells in the GC007F cell population. These results showed that GC007F cells exhibited a potent killing capacity in vitro.

### GC007F cells have a potent in vivo killing capacity and high persistence

The tumor burdens in the high-dose and medium-dose groups were diminished 11 days after infusion, and the mice in these groups remained in CR with further decreases in their tumor burdens observed at 21 days after infusion, but the medium-dose group exhibited a slightly higher tumor burden than the high-dose group (Fig. 3E). When GC007F and C-CAR-T cells were directly compared, C-CAR-T cell infusion delayed tumor progression, but dead mice were found on day 16, and no mice survived more than 37 days. Tumors were well controlled in the GC007F cell-treated group, and no mice died before day 42 (Fig. 3F, G). In the control mouse groups without T cell infusion (vehicle) or with infusion of nontransduced (C-NT or F-NT) cells, tumors grew progressively.

GC007F cells also expanded robustly after infusion into tumor-bearing mice (Fig. 3H). On day 28 after infusion, GC007F and C-CAR-T cell numbers peaked. In the peripheral blood, 123.2 GC007F cells/µl were detected, but only 16.7 cells/µl were detected in the C-CAR-T cell-infused group. Significant differences were found on days 14 and 21 after CAR-T cell infusion (*P* = 0.0479). On day 40 after CAR-T cell infusion, no C-CAR-T cells were detected, but GC007F cells persisted in the peripheral blood.

### GC007F FasT-CART demonstrated preliminary favorable efficacy results in patients with R/R B-ALL including a high CR rate

A total of 21 patients were screened, enrolled and infused with a single infusion of GC007F cells. The median CAR positive rate was 39.5% (15.1% ~ 60.4%). The release criteria for GC007F cells was that the CAR positive rate was ≥10%. Three patients gave up early within 2 months after CAR-T cell infusion. Eighteen patients were available for response assessment. One patient gave up on day 6 due to septic shock and respiratory failure, and another patient gave up on day 9 due to intracranial hemorrhage. A third patient was noncompliant with treatment and experienced severe myelosuppression and infection after CAR-T cell infusion; he gave up on day 44. By day 28, all efficacy-evaluable patients (18/18, 100%) had achieved CR, and 17 of the 18 patients (94.4%) achieved CR with MRD negative. Sixteen of the patients (83.3%) maintained CR at day 84, while 14 patients (77.8%) maintained MRD negative. Patient characteristics are shown in Table 1.

**Table 1:** Clinical study patient characteristics (*n* = 21).

Pt	Sex	Age	Disease	Cyto genetics	Chemotherapy cycles	Pretreatment Regimen	BM Blast pre-lymphodepletion (%)	GC007F cell dose	CAR^+^ (%)	CRS	ICANS	CR
1	F	39	Ph^+^ B-ALL	T315I/V299L	5	VDLP + TKI×2, CAM×1 HyperCVAD (Part B) + TKI × 2	0.71	DL1	31.8	1	No	MRD^-^
2	F	14	Ph^-^ B-ALL	Normal	3	VDLP × 1, CAM × 1 HyperCVAD (Part B) × 1	0.05	DL1	33.30	No	No	MRD^-^
3	F	27	Ph^-^ B-ALL	Normal	4	CVDLP × 2 HyperCVAD (Part B) × 2	87.87	DL1	32	3	No	MRD^-^
4	F	27	Ph^+^ B-ALL	CALR, T315I	5	VDLP + TKI×3 CAM + TKI×1 HyperCVAD (Part B) + TKI × 1	85.47	DL2	40.1	2	No	MRD^+^
5	M	25	Ph^+^ B-ALL	BCR/ABL	3	CVDLP + TKI × 2 HyperCVAD (Part B) + TKI × 1 Allogeneic transplant	47.86	DL2	15.1	3	No	MRD^-^
6	F	21	Ph^-^ B-ALL	Normal	4	VDLP × 2 HyperCVAD (Part B) × 2	0.05	DL2	19.1	3	No	MRD^-^
7	M	15	Ph^+^ B-ALL	BCR/ABL	5	VDLP + TKI × 2 CAM + TKI × 1 HyperCVAD (Part B) + TKI × 2	0.45	DL2	46.1	3	No	MRD^-^
8	M	15	Ph^-^ B-ALL	Normal	3	VDLP × 1 HyperCVAD (Part B) × 2	7.27	DL2	33.7	3	3	MRD^-^
9	M	24	Ph^-^ B-ALL	TEL/AML	5	CVDLP × 3 HyperCVAD (Part B) × 2	94.01	DL2	42	2	3	MRD^-^
10	F	61	Ph^-^ B-ALL	IKZF1,SH2B3	5	VDLP + TKI × 3 HyperCVAD (Part B) + TKI × 2	0.01	DL2	26.1	2	No	MRD^-^
11	M	47	Ph^-^ B-ALL	Normal	2	CVDLP × 1, CAM × 1	59.74	DL2	42.2	2	No	MRD^-^
12	F	41	Ph^-^ B-ALL	Normal	2	VDLP × 2	57.14	DL2	39.5	3	No	MRD^-^
13	M	17	Ph^-^ B-ALL	Normal	7	VDLP × 4, CAM × 2 HyperCVAD (Part B) × 1	17.86	DL3	46.9	3	2	MRD^-^
14	F	18	Ph^-^ B-ALL	E2A-PBX1	5	VDLP × 2, CAM × 1 HyperCVAD (Part B) × 2 Allogeneic transplant	1.77	DL3	50.9	3	1	MRD^-^
15	M	32	Ph^-^ B-ALL	Normal	5	CVDLP × 2, CAM × 1 HyperCVAD (Part B) × 2	69	DL3	46	2	No	MRD^-^
16	F	14	Ph^-^ B-ALL	WTI	5	CVILP × 2, CAM × 1 HyperCVAD (Part B) × 2	1.67	DL3	40.1	3	3	MRD^-^
17	F	44	Ph^-^ B-ALL	Normal	5	CVILP × 2, CAM × 1 HyperCVAD (Part B) × 2 Allogeneic transplant	38.5	DL3	60.4	2	No	MRD^-^
18	M	47	Ph^-^ B-ALL	Normal	9	CVDLP × 2, CAM × 1 HyperCVAD (Part B) × 2, et al.	85.29	DL3	39.4	3	No	MRD^-^
19	F	32	Ph^-^ ALL	MLL-AF4	2	VDLP × 1 HyperCVAD (Part B) × 1	87.31	DL2	26.8	2	2	NE
20	M	44	Ph^-^ ALL	Normal	5	CVDLP × 2, CAM × 1 HyperCVAD (Part B) × 2	62	DL2	21.3	4	No	NE
21	M	45	Ph^-^ ALL	Normal	25	CVDLP × 3, CAM × 1, HyperCVAD (Part B) × 2, et al.	92.3	DL3	43.3	3	No	NE

*Pt* Patient, *CAR-T cell* chimeric antigen receptor T cell, *CRS* cytokine release syndrome, *ICANS* immune effector cell-associated neurotoxicity syndrome, *CR* complete remission, *MRD* minimum residual disease, *Ph*^*+*^*-B-ALL* Philadelphia chromosome positive acute lymphoblastic leukemia, *Ph*^*-*^*-B-ALL* Philadelphia chromosome negative acute lymphoblastic leukemia, *BM* bone marrow, *VDLP* vincristine, daunorubicin, L-asparaginase and prednisone, *TKI* Tyrosine Kinase Inhibitor, *HyperCVAD* (Part B): dexamethasone, methotrexate and cytarabine, *CAM* cyclophosphamide, cytarabine, 6-mercaptopurine, *CVDLP* cyclophosphamide, vincristine, daunorubicin, L-asparaginase and prednisone, *CVILP* cyclophosphamide, vincristine, idarubicin, L-asparaginase and prednisone, *NE* Not evaluable.

### GC007F cell pharmacokinetics in patients

**Fig. 4:**
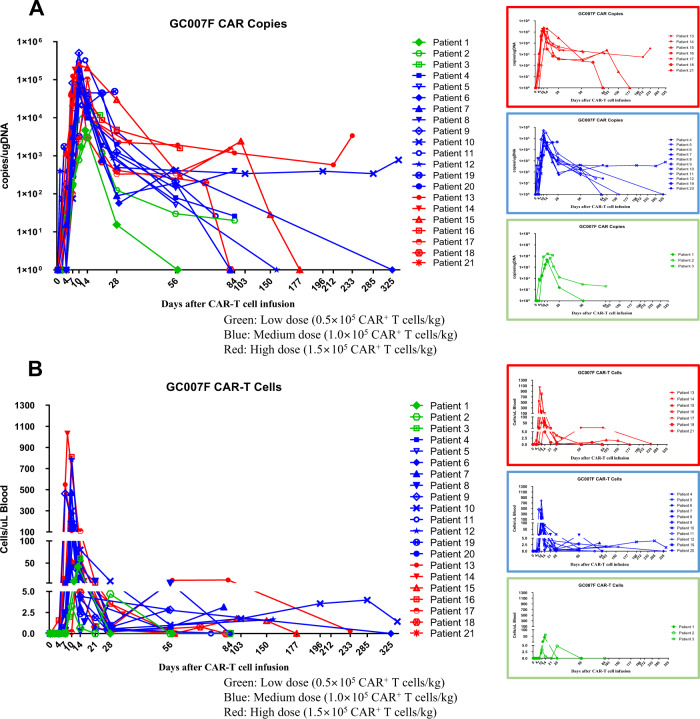
The persistence of CAR-T cells in the peripheral blood of patients enrolled in the clinical study (*n* = 21) was evaluated using qPCR and flow cytometry. Peripheral blood mononuclear cells (PBMCs) were obtained from enrolled patients with R/R B-ALL. The patients underwent conditioning according to their disease burden. GC007F cells were infused after 24–48 h of conditioning. After GC007F cell infusion, PBMCs were collected to analyze cell pharmacokinetics on days 4, 7, and 10 and weeks 2, 4, 8 and 12. Three doses of GC007F cells (dose level (DL) 1 (green): 0.5 × 10^5^ CAR^+^ T cells/kg, DL2 (blue): 1.0 × 10^5^ CAR^+^ T cells/kg, and DL3 (red): 1.5 × 10^5^ CAR^+^ T cells/kg) were used in this study. **A** The CAR copies/µg DNA of GC007F cells was detected using qPCR. **B** The numbers of CAR^+^ (GC007F) cells were detected using flow cytometry.

GC007F cell numbers detected by qPCR peaked on day 10 (range: days 7–27) after infusion. The median persistence period of GC007F cells in the peripheral blood was 56 days (range: 7–327 days) after infusion. The longest persistence period was 11.7 months and was ongoing at the last follow-up time point. The median peak CAR copy number was 104899.5/µg DNA (range: 614–504158/µg DNA) (Fig. 4A and Table S1).

GC007F cell numbers detected by flow cytometry peaked on day 10 (range: days 7–28) after infusion. The median persistence period of CAR-T cells in the peripheral blood was 56 days (range: 7–327 days) after infusion. The longest persistence period was 11.7 months and was still ongoing at the last follow-up time point. The median peak cell number was 158 cells/µl blood (range: 0.32–1031 cells/µl blood) (Fig. 4B and Table S1).

### Cytokine release in patients

For all patients, the peak levels of IL-6, IL-10, tumor necrosis factor (TNF)-α, IFN-γ and granulocyte-macrophage colony-stimulating factor (GM-CSF) were 209.56 pg/mL, 147.485 pg/mL, 16.51 pg/mL, 656.48 pg/mL, and 24.89 pg/mL, respectively (Fig. S1). All levels peaked on day 7. The time of the peak levels for IL-1α, IL-1β, IL-2, IL-7, IL-8, IL-12p70, IL-15, granzyme B, CCL19, CCL3, CCL4, monocyte chemotactic protein (MCP)-1, vWF-A2, and angiopoietin-2 was also day 7. The peak level times for C-reactive protein (CRP), angiopoietin-1, and tumor necrosis factor-related apoptosis-inducing ligand (TRAIL) were 14, 21 and 28 days, respectively.

### Safety

Overall, CRS of any grade occurred in 20 of the 21 patients (95.2%), and CRS grade ≥3 occurred in 11 patients (52.4%). Six patients (28.6%) developed neurotoxicity, with 3 patients (14.3%) grade ≥3. All patients recovered after treatment with Standard of Care. The abnormal results of laboratory tests are shown in Table S2 in the Supplementary information.

The incidence of CRS was not related to the CAR-T cell number or disease burden (*P* = 0.197 and *P* = 0.312, respectively). Severe CRS was also not related to the CAR-T cell number or disease burden (*P* = 0.271 and *P* = 0.568, respectively). We did not observe a relationship between neurotoxic effects and the CAR-T cell number or disease burden (*P* = 0.471 and *P* = 0.702, respectively). Severe neurotoxic effects were also not related to the CAR-T cell number or disease burden (*P* = 0.814 and *P* = 0.69, respectively).

Univariate analysis showed that the TRAIL level was related to the disease burden (*P* = 0.037), IL-2 was related to the infused dose of CAR-T cells (*P* = 0.002), and TNF-α, IL-6, IL-10, IFN-γ, IL-2, GM-CSF, and granzyme B were related to severe manifestations of CRS (*P* = 0.003, *P* = 0.001, *P* = 0.019, *P* = 0.002, *P* = 0.009, *P* = 0.005, and *P* = 0.015, respectively). No cytokines were related to immune effector cell-associated neurotoxicity syndrome. Multivariate analysis showed that no cytokines were related to severe manifestations of CRS.

The occurrence of B cell aplasia and hypogammaglobulinemia was common, with hypogammaglobulinemia occurred in 16 of the 21 patients (76.19%). B cells became undetectable in all patients achieving CR regardless of their MRD status. Intravenous immunoglobulin repletion dosed according to patient immunoglobulin levels was used to manage hypogammaglobulinemia. B cell aplasia was sustained for all patients with continuous CR, further indicating that the persisting GC007F cells remained functional.

### Efficacy results

**Fig. 5:**
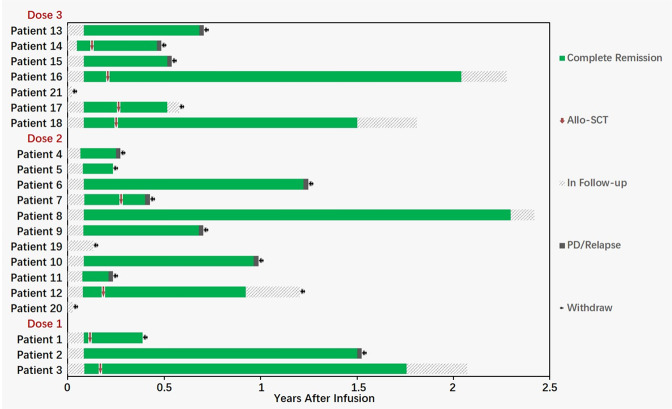
Data for survival and use of allo-HSCT after CAR-T cell treatment for patients with R/R B-ALL enrolled in the clinical study (*n* = 21). All 18 efficacy-evaluable patients (100%) were followed for at least 3 months; 15 of these patients (83.3%) maintained disease-free survival (DFS) at the 3-month assessment, and 14 patients (77.8%) remained MRD negative at 3 months. The longest time of disease-free survival was 29 months.

All 18 efficacy-evaluable patients were followed for at least 3 months; 15 of these patients (83.3%) were disease free at the 3-month assessment, and 14 (77.8%) maintained MRD negative at month 3 (Fig. 5). The longest duration of response to date is 29 months without transplant.

Eight of the 18 patients underwent allogenic hematopoietic stem cell transplantation (allo-HSCT) after GC007F cell treatment. Of the patients who underwent allo-HSCT, 2 patients relapsed at months 4.8 and 6, 1 gave up treatment due to severe infection and respiratory failure during transplant period, 1 was diagnosed with posttransplant lymphoproliferative disorder (PTLD) and received other treatment, 1 gave up treatment due to graft-versus-host disease (GvHD) caused by transplant, and the other 3 patients still maintained MRD- CR. The longest time of disease-free survival following allo-HSCT was 27.3 months.

For the 10 patients who did not proceed to transplant, 8 patients relapsed (7 with marrow, 1 with extramedullary), 1 died of severe infection and septic shock on day 83, and 1 maintained MRD- CR up to the last follow-up. The longest time of disease-free survival among the patients who did not proceed to transplant was 29 months.

## Discussion

The clinical application of CAR-T cells is undoubtedly a milestone in the history of cancer treatment. The CR rate of CD19-directed CAR-T cells in the treatment of R/R B-ALL has reached 67–93%[19, 20]. However, the long wait time for manufacturing, high relapse rate, toxicity and side effects remain problematic. Our study showed that GC007F cells had a superior expansion capacity and a younger and less exhausted phenotype than C-CAR-T cells. Our first-in-human clinical study showed that novel GC007F cells were efficacious with a manageable toxicity profile in the treatment of R/R B-ALL.

A cellular product with a shorter manufacturing time and larger proportion of younger T cells may significantly enhance effector function and proliferative capacity. Previously, some methods have been used to prevent the differentiation that occurs during T cell expansion, such as Akt inhibition or blockade of Fas-FasL interactions [21, 22]. However, the application of these approaches during long-duration culture is cost- and labor-intensive, which prohibits the widespread clinical application of this technology [23]. The current routine methods for the production of C-CAR-T cells require 9–14 days of ex vivo culture. [13] Previous studies have shown that relatively high amounts of T_CM_, memory and naïve T cells in culture are related to long-term persistence in vivo [24–30]. Only one day was required for the construction of novel GC007F cells with a shorter manufacturing time, decreased costs and reduced labor needs [31–33]. Notably, the GC007F cells evaluated in this study had a relatively high T_CM_ cell proportion, and the abilities to proliferate, secrete effector cytokines and antitumor activity were enhanced in our studies [34]. The inhibitory receptors PD-1, LAG3 and Tim3 were expressed on relatively few GC007F cells in the current study, which suggested the potential for high potency and longer-term persistence of GC007F cells in clinical applications. Increased proliferative and cytolytic abilities were also observed in vitro and in animal models. Overall, the preclinical study showed that GC007F cells had a shorter culture time with a superior expansion capacity, a younger and less exhausted phenotype and better killing abilities than C-CAR-T cells.

Long-term C-CAR-T cell culture can result in an increase in the disease burden and progression during the waiting period to the point where patients are not eligible for CAR-T cell treatment [9, 10]. Even in patients who are able to receive C-CAR-T cell infusion, the increased disease burden also leads to worse outcomes and severe adverse events. All patients in the present study successfully and quickly received infusion of our novel GC007F cells. Cell quantity is also important in CAR-T cell treatment. Relatively low CAR-T cell doses sustainably controlled leukemia in a xenograft model in the current study. GC007F cells administered at a low dose (0.5 × 10^5^) also exhibited enhanced proliferation, effector function, and persistence in our clinical study. The median persistence time was 56 days, with a peak copy number of 104899.5/µg DNA in patients, and this time is longer than that reported for C-CAR-T cells [9, 10]. Future studies should investigate much lower doses of GC007F cells for R/R ALL. The incidence of CRS was high in this study, even with infusion of a low dose, which may be related to the quicker proliferation and high proliferative potential of GC007F cells. Recent studies have shown that early intervention with steroids and tocilizumab does not impact the expansion, persistence or efficacy of CAR-T cells [35]. Therefore, the early or preventive use of drugs may decrease toxicity without compromising the efficacy of GC007F cells in future studies.

It should also be noted that, the expanded clinical application of FasTCAR platform needs to be further evaluated. For GC007F cells there are more studies needed to determine dose based on current findings. Larger studies are needed to further evaluate the efficacy including PFS and OS. The risk benefit needs to be further characterized including evaluation of preventive strategies to manage CRS. Lastly also duration of response needs to be further evaluated in studies to determine follow in strategies in case of MRD relapse.

In summary, our results demonstrate that compared with C-CAR-T cells, GC007F cells produced with the novel FasTCAR platform have a superior expansion capacity and a younger phenotype and are less exhausted. Data generated show preliminary efficacy results in patients with R/R B-ALL that are promising and need further evaluation. Moreover, this first-in-human clinical study, conducted in patients with R/R B-ALL in China, showed that CD19-directed GC007F cell therapy were effective in eliminating CD19 positive B-ALL with a manageable toxicity. The safety, efficacy, and potential long-term clinical benefits of GC007F cell therapy warrant further clinical evaluation.

## Supplementary information

Supplementary information

## Data Availability

The datasets generated and/or analyzed during the current study are available from the corresponding author on reasonable request.
