# Supplementary material for: Novel CD19 chimeric antigen receptor T cells manufactured next-day for acute lymphoblastic leukemia
Source: Blood Cancer J. 2022 Jun 24;12(6):96. doi: 10.1038/s41408-022-00688-4 (PMC9232607; doi:10.1038/s41408-022-00688-4)
Supplement: Supplementary file 1 — Supplementary information [file 41408_2022_688_MOESM1_ESM.pdf]

## Supplementary information for

### Novel CD19 Chimeric Antigen Receptor (CAR) T Cells Manufactured Next-day for Acute Lymphoblastic Leukemia (ALL)

Cheng Zhang[1*], Jiaping He[2*], Li Liu[3], Jishi Wang[4], Sanbin Wang[5], Ligen Liu[6], Jian Ge[7], Lei Gao[1], Li Gao[1], Peiyan Kong[1], Yao Liu[1], Jia Liu[2], Yu Han[2], Yongliang Zhang[2], Zhe Sun[2], Xun Ye[2], Wenjie Yin[2], Martina Sersch[2], Lianjun Shen[2#], Wei William Cao[2], Xi Zhang[1#]

[1]Medical Center of Hematology, Xinqiao Hospital, State Key Laboratory of Trauma, Burn and Combined Injury, Army Medical University, Chongqing, P.R. China.

[2]Gracell Biotechnologies Ltd., Shanghai, P.R. China.

[3]Department of Hematology, Tangdu Hospital, Air Force Medical University, Xi'an, Shaanxi, P.R. China.

[4]Department of Hematology, The Affiliated Hospital of Guizhou Medical University, Guiyang, Guizhou, P.R. China.

[5]Department of Hematology, 920th Hospital of Joint Logistics Support Force, Kunming, Yunnan, P.R. China.

[6]Department of Hematology, Tongren Hospital, Shanghai Jiao Tong University School of Medicine, Shanghai, P.R. China.

[7]Department of Hematology,The First Affiliated Hospital of Anhui Medical University, Hefei, Anhui, P.R. China.

**Cell lines**

Four cell lines were used to evaluate and compare the tumor killing capacity of GC007F cells and C-CAR-T cells. K562 (human chronic myelogenous leukemia) (ATCC, CCL-243™), Raji (B cell Burkitt's lymphoma) (ATCC, CCL-86™), NALM6 (B cell leukemia) (ATCC CRL-3273), and HeLa (cervical cancer) (ATCC, CCL-2) cells were purchased from ATCC. The cell lines were cultured and maintained in RPMI-1640 medium (Invitrogen, Carlsbad, CA, USA) supplemented with 10% heat-inactivated FBS (Invitrogen) at 37 °C and 5% $CO_2$. CD19-expressing K562 (K562-CD19) and HeLa (HeLa-CD19) cells were generated by lentiviral transduction with a vector purchased from GeneChem. For *in vitro* and *in vivo* tumor cell-killing assays, Raji and NALM6 cells were also transduced with luciferase using a lentivirus purchased from GeneChem.

**Antibodies**

Commercial antibodies were purchased for use in the flow cytometric analyses performed in the preclinical and clinical portions of this study. The following anti-human antibodies were obtained from eBioscience: FITC-conjugated anti-CD3 (OKT 3, Cat# 340542) and APC-conjugated anti-LAG-3 (Cat# 17–2239–42). The following antibodies were purchased from BioLegend: PE-conjugated anti-DYKDDDDK (Cat# 637310) and BV421-conjugated anti-PD-1 (Cat# 329920). The following antibodies were purchased from BD Bioscience: FITC-conjugated anti-CD4 (Cat#340133), APC-H7-conjugated anti-CD8 (Cat# 560179), BV510-conjugated anti-CD45RA (Cat# 563031), BV421-conjugated anti-CD62L (Cat# 563862), APC-conjugated anti-CD45RO (Cat# 340438), and PE/Cy7-conjugated anti-human CD184 (CXCR4) (Cat# 306514). A 7-AAD solution (Cat# 51–68981E) was also purchased from BD Bioscience.

**Flow cytometry**

For blood samples collected from patients (clinical study) or animals (preclinical studies), 100-µl peripheral blood samples were aliquoted into EDTA tubes. Erythrocytes were lysed for 8 minutes at room temperature in a red blood cell lysis solution (BD Bioscience, 349202), and the white blood cells were centrifuged at 300 g for 5 minutes at room temperature and washed

with 2 ml of PBS before staining. Human T cells were identified by surface staining for CD45 and CD2. CD19-CAR expression was detected using an anti-FLAG antibody. For deep immunophenotyping of T cells, CAR-T cell products were harvested, washed twice with 2 ml of PBS, surface stained with commercially available flow cytometry antibodies at 4 °C for 30 minutes, and washed twice with 2 ml of PBS. All flow cytometry antibodies were titrated according to the manufacturer's instructions before use, and fluorescence minus one controls were created for each antibody panel to set gates for positive events. 7-AAD and absolute counting beads were added, and samples were run on a FACSCanto flow cytometer for 2 hours. Data were analyzed using FlowJo software. In the clinical study, peripheral blood was collected at different time points to evaluate CAR-T cell pharmacokinetics.

**qPCR**

Cellular genomic DNA (gDNA) was extracted from 1 ml of whole blood using the QIAamp DNA Blood Midi Kit (Qiagen, Redwood City, CA, United States, 51185). One hundred nanograms of DNA was amplified using the TB Green Premix ExTaq (Tli RnaseH Plus) Kit (Takara Biotechnology, Dalian, China, RR420A) with the corresponding amplification primer pairs, premixed TB Green Premix Ex TaqII (Tli RnaseH Plus) solution and rOX reference dye following the manufacturer's recommended protocols. Amplification was detected in real time using the Applied Biosystems 7500 Real-Time PCR System (Life Technologies, MA, USA). A primer pair targeting the WPRE region was used to measure the CAR copy number, and a primer pair targeting the RPP30 gene was used as a reference. The primer pairs were experimentally validated using the following criteria: (i) a single gene-specific product was produced; (ii) the amplification efficiency ranged between 90% and 110%; and (iii) the cycle threshold (Ct) value of the no-template control was greater than 40.

Primers: WPRE F, 5'-TTTGTGAAAGATTGACTGGTATTCT-3';

R, 5'-AAAGGCATTAAAGCAGCGTATC-3';

RPP30 F, 5'-CAGCTTCCAAGAAAGCCAAGTG-3';

R, 5'-GTGGCTGATGAACTATAAAAGGGA-3'.

The preliminary CAR copy number was calculated after comparing the corresponding Ct value

and the standard curve Ct value generated by serial dilution of plasmid standard samples into 100 ng of background human gDNA. An adjustment factor was derived from RPP30 gene amplification results in controls and samples, $AF=2^{-(Ct\ RPP30(Control)-\ Ct\ RPP30(Sample))}$. The final CAR copy number was calculated as preliminary CAR copy number × AF × {1 g/100 ng}.

**Cytokine assay**

For the real-time cell analysis (RTCA) cytotoxicity assay for cultured cells, cell supernatants were harvested and frozen at −70 °C immediately after centrifugation at 4 °C and 300 g for 5 minutes. The production of the cytokines interferon-γ (IFN-γ) and interleukin (IL)-2, as markers of T cell killing and activation, respectively, was quantified using customizable assays on the Ella platform (Protein Simple).

To detect serum cytokine production in GC007F cell-treated patients, human blood samples were collected at various time points after GC007F cell infusion. After centrifugation at 4 °C and 1000 g for 10 minutes, serum samples were aliquoted and immediately frozen at −70 °C. Cytokines were analyzed in duplicate after 2-fold dilution. Briefly, 50 μl of suspended magnetic beads and 50 μl of sample were loaded into each plate well. After 2 hours of incubation with gentle shaking at 800 rpm, the plate was washed 3 times under a magnetic frame. The beads were further incubated with 50 μl of biotinylated multiplex antibody for 1 hour with gentle shaking at 800 rpm and then washed accordingly. The beads were incubated with 50 μl of streptavidin-PE for 1 hour with gentle shaking at 800 rpm and washed accordingly. Finally, the beads were suspended in 100 μl of buffer, and the plate was read on a Luminex 200 apparatus (Luminex Corporation, MN, USA) according to the manufacturer's instructions.

The levels of the following cytokines, chemokines and growth factors were measured in clinical samples collected on days 4, 7, and 10 and weeks 2, 4, 8 and 12: IL-1α, IL-1β, IL-2, IL-6, IL-7, IL-8, IL-10, IL-12p70, IL-15, tumor necrosis factor α (TNF-α), granulocyte-macrophage colony-stimulating factor (GM-CSF), IFN-γ, granzyme B, CCL19, CCL3, CCL4, monocyte chemotactic protein 1 (MCP-1), C-reactive protein (CRP), tumor necrosis factor-related apoptosis-inducing ligand (TRAIL), vWF-A2, angiopoietin-1, and angiopoietin-2.

### *In vivo* animal model

For the dose-dependent efficacy study, NOG mice (6-10w, female) were purchased form the Vital River Laboratory (Beijing, China). The NOG mice were injected intravenously (i.v.) with Nalm6-Luciferase cells ($1\times10^6$/mouse), followed by intravenous infusion of various doses of CD19-targeted CAR-T cells ($1\times10^5$, $5\times10^5$, or $1\times10^6$ CAR-T cells/mouse, 10 mice/group) 7 days later. Tumor growth in the Nalm6-Luciferase cell-inoculated mice was monitored by bioluminescence imaging (BLI) using an In Vivo Ms Fx Pro by Bruker (USA) once weekly for three weeks. The tumor burden was evaluated using quantitative analysis of the bioluminescence signal intensity on days 0, 5, 8, 13, 15, 19, 22, 26, 29, 35, and 42 after CAR-T cell infusion.

For the survival studies that also evaluated the tumor burden, NOG mice were i.v. inoculated with $3\times10^5$ Raji-Luciferase tumor cells 6 days before intravenous injection of $5\times10^5$ CAR-T cells. Tumor growth in the Raji-Luciferase cell-inoculated mice was monitored by BLI using a Lumina III (Perkin Elmer, USA) twice weekly for four weeks and then once weekly for two weeks. The tumor burden was evaluated via quantitative analysis of the bioluminescence signal intensity using Living Image v 4.5 by PerkinElmer. Cells were acquired on a BD FACSCanto II (BD, USA) and analyzed with FlowJo X (BD, USA).

**Table S1**. GC007F cell persistence at different dose levels (n=21).

| | qPCR | | | | FCM | | | |
|---|---|---|---|---|---|---|---|---|
| | **Overall** | **DL1** | **DL2** | **DL3** | **Overall** | **DL1** | **DL2** | **DL3** |
| **Peak time (day)** | 10 (7-27) | 14 (13-14) | 10 (7-27) | 10 (8-14) | 10 (7-28) | 14 (13-28) | 10 (7-11) | 10 (7-14) |
| **Persistence period (day)** | 56 (7-327) | 28 (14-84) | 56 (7-327) | 76 (34-233) | 56 (7-327) | 14 (13-28) | 56 (7-327) | 52 (28-233) |
| **Peak number** | 104899.5/μg DNA (614-504158) | 6643/μg DNA (4670.2-16008) | 73941/μg DNA (614-504158) | 149828.15/μg DNA (102847-247507.1) | 158 cells/μl blood (0.32-1031) | 42.4 cells/μl blood (4.7-60.9) | 161 cells/μl blood (0.32-787.2) | 360 cells/μl blood (60.8-1031) |

Notes: qPCR: Quantitative real-time PCR; FCM: Flow cytometry; DL: Dose level. DL1: 0.5 x $10^5$ $CAR^+$ T cells/kg, DL2: 1 x $10^5$ $CAR^+$ T cells/kg, and DL3: 1.5 x $10^5$ $CAR^+$ T cells/kg.

**Table S2**. Grades of adverse events by laboratory tests after GC007F cell treatment (n=21).

| Pt | APTT ↓ | Fib ↓ | ALP ↑ | ALT ↑ | AST ↑ | LDH ↑ | GGT ↑ | Bilirubin ↑ | WBC ↓ | ANC ↓ | TLC ↓ | PLT ↓ | CMV infection | Pulmonary infection | Pyemia |
|---|---|---|---|---|---|---|---|---|---|---|---|---|---|---|---|
| 1 | | | | | | | | | 4 | 4 | 4 | | | | |
| 2 | | | | | | | | | 3 | 3 | 4 | | | | |
| 3 | | | | 2 | 2 | 1 | | | 4 | 4 | 4 | 4 | | | |
| 4 | 1 | 1 | | 1 | | | 3 | | 3 | 3 | 4 | 4 | | | |
| 5 | 1 | 1 | 3 | 1 | 1 | | | 3 | 4 | 4 | 4 | 1 | 3 | 5 | 4 |
| 6 | | 4 | | | 2 | | | | 4 | 4 | 4 | 4 | | | |
| 7 | | 2 | | | | 1 | | | 3 | 2 | 4 | | | | |
| 8 | | | | | | 1 | 3 | 2 | 4 | 4 | 4 | 1 | | | |
| 9 | | 2 | | 2 | 2 | 1 | 3 | | 4 | 4 | 4 | 4 | | | |
| 10 | 1 | 2 | | 2 | 2 | 1 | 3 | 2 | 4 | 4 | 4 | | | 2 | |
| 11 | | | | | | | | | 4 | 4 | 4 | 4 | | | |
| 12 | | 3 | 2 | | | | | 3 | 4 | 4 | 4 | 4 | | 3 | |
| 13 | 1 | 2 | | 1 | 1 | 1 | | 1 | 4 | 4 | 4 | 3 | | | |
| 14 | | 4 | | 1 | 3 | 1 | 2 | | 3 | 3 | 3 | 4 | | | |
| 15 | | 3 | | | | 1 | 3 | 1 | 4 | 4 | 4 | | | | |
| 16 | | 3 | | | 2 | 1 | | | 4 | 4 | 4 | 4 | | | |
| 17 | 1 | 1 | | | | 1 | | | 4 | 4 | 3 | 4 | | 3 | |
| 18 | 1 | 2 | | | | | | | 4 | 4 | 4 | 4 | | 3 | |
| 19 | | 3 | | 2 | 3 | 1 | 3 | | | | | | | | |
| 20 | | | | | | | | 1 | 4 | 4 | | 4 | | | |
| 21 | | | | | 2 | | | | | | | | | 4 | |

Notes: Pt: Patient; APTT: Activated partial thromboplastin time; Fib: Fibrinogen; ALP: Alkaline phosphatase; ALT: Alanine aminotransferase; AST: Aspartate aminotransferase; LDH: Lactate dehydrogenase; GGT: Gamma glutamine transferase; WBC: White blood cell count; ANC: Absolute neutrophil count; TLC: Total lymphocyte count; PLT: Platelet count; CMV: Cytomegalovirus.

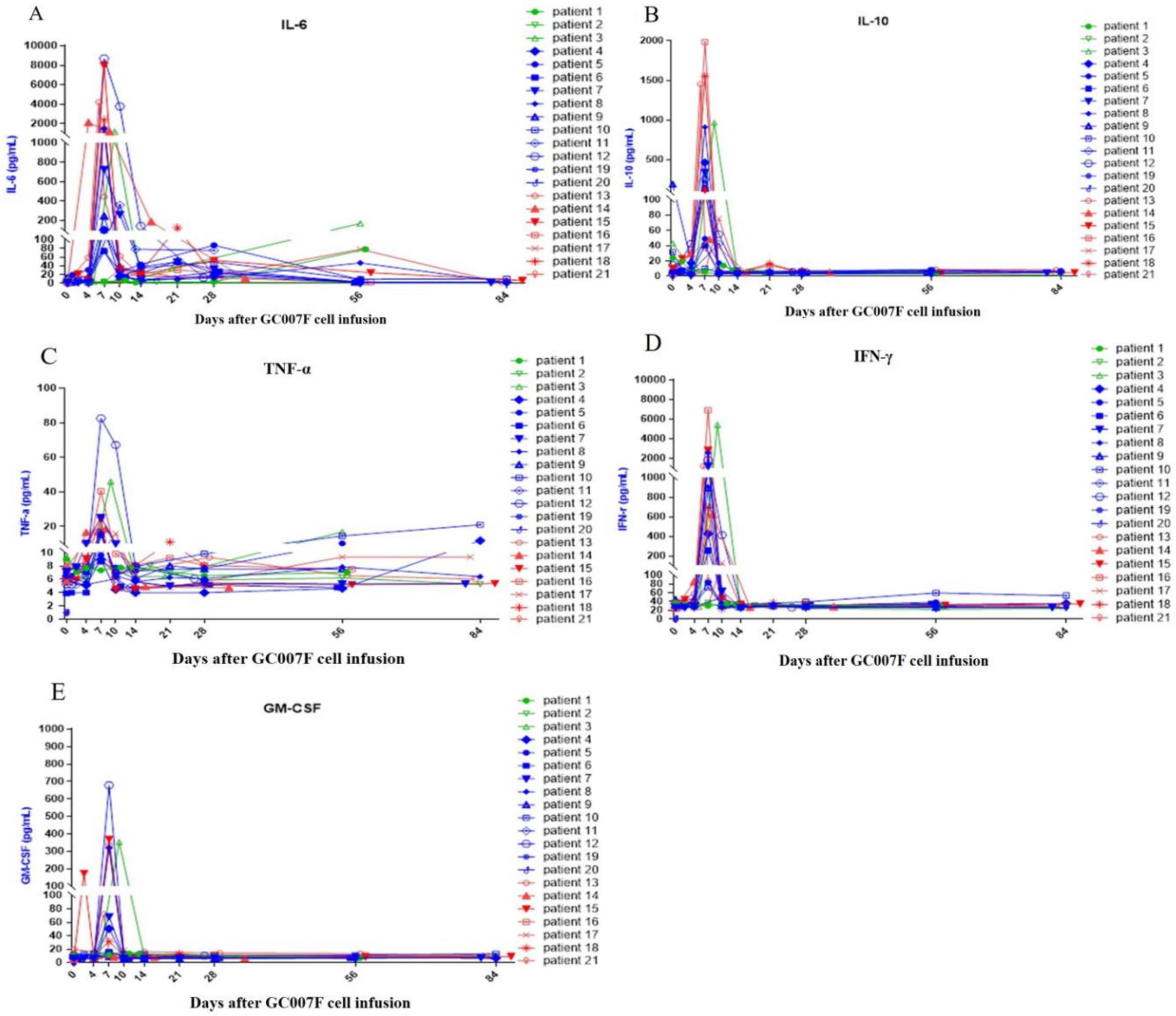


**Fig. S1**. The expression of cytokines, chemokines and growth factors was detected at different time points after CAR-T cell administration infusion. Factors were detected using enzyme-linked immunosorbent assay after GC007F cell transfusion at different time points. The levels of the main factors shown peaked on day 7. (A) IL-6. (B) IL-10. (C) TNF-α. (D) IFN-γ. (E) GM-CSF. Notes: IL-6: Interleukin 6; IL-10: Interleukin 10; TNF-α: Tumor necrosis factor α; IFN-γ: Interferon-γ; GM-CSF: Granulocyte-macrophage colony-stimulating factor.
